# T-bet and Eomesodermin in NK Cell Development, Maturation, and Function

**DOI:** 10.3389/fimmu.2016.00241

**Published:** 2016-06-20

**Authors:** Federico Simonetta, Amandine Pradier, Eddy Roosnek

**Affiliations:** ^1^Department of Medical Specialties, Division of Hematology, Geneva University Hospitals, University of Geneva, Geneva, Switzerland

**Keywords:** natural killer cells, T-box transcription factors, T-bet, Eomes

## Abstract

Recent reports give insights into the role of the T-box transcription factors, T-bet and Eomesodermin (Eomes), in NK cell biology. In this mini-review, we recapitulate the initial reports that delineate T-bet and Eomes as master regulators of NK cell development, maturation, and function. We discuss how T-bet and Eomes expression is regulated during NK cell development and peripheral maturation. Furthermore, we summarize the current literature on the role of T-bet and Eomes in the transcriptional regulation of NK cell function and review possible effects of T-box transcription factor anomalies during aging, infection, cancer, and after hematopoietic stem cell transplantation. We discuss how the current data argue in favor of a model of T-bet and Eomes synergy in transcriptional regulation of NK cell function and identify T-box transcription factors as potential targets for therapeutic interventions.

## Introduction

The phylogenetically conserved family of T-box transcription factors, which share T-box DNA-binding domains, is critically involved in developmental processes in vertebrates. The T-box protein in T cells (T-bet) is a tyrosine- and serine-phosphorylated protein encoded by the *Tbx21* gene that is expressed only in cells of hematopoietic origin. T-bet was originally identified in T lymphocytes as the key transcription factor involved in interferon-gamma (IFN-γ) production that commits CD4 T cells to the Th1 lineage (1). Eomesodermin (Eomes), another T-box transcription factor sharing homology with T-bet, was originally described as a key player in vertebrate embryogenesis (2). More recently, Eomes and T-bet have been reported to coordinate the differentiation of CD8 T cells into effector cells (3–5) as well as their transition to the memory cell pool (6, 7). T-bet and Eomes are therefore considered as master regulators of T cell function. The bulk of mature murine (6, 8, 9) and human (10–12) NK cells express high levels of T-bet and Eomes, but until recently, their impact on NK cell function was not known. In the present work, we summarize the current knowledge about the role of T-bet and Eomes in NK cell development, peripheral maturation, and function.

## T-bet and Eomes in NK Cell Development

The first evidence for a role of T-bet in NK cell biology came from the observation that T-bet deficient (T-bet^−/−^) mice have slightly higher NK cell numbers in the bone marrow but reduced numbers of NK cells in spleen, liver, and peripheral blood (13). Because many NK cells in T-bet^−/−^ mice expressed an immature CD27^pos^CD11b^pos^ phenotype, it was suggested that T-bet played a role in NK cell maturation without being essential for the early stages of NK cell development. Because Eomes^−/−^ mice die in an early embryonic stage, the role of Eomes in NK cell development has initially been assessed only in compound mutant Eomes^+/−^ Tbx21^−/−^ mice (6). Interestingly, Eomes^+/−^ Tbx21^−/−^ mice displayed a severely exacerbated defect in the NK cell compartment compared to mice only lacking T-bet, suggesting a distinct, but complimentary function for Eomes in NK cell development. Importantly, the loss of one allele of *Eomes* results in a severe downregulation of CD122 (6), the beta-chain of IL-2R and IL-15R, which is essential for IL-15 signaling and NK cell development. Chromatin Immunoprecipitation (ChIP) assays showed that Eomes regulated CD122 transcription (6) for which T-bet appeared to be unnecessary (13). More recently, the role of Eomes has been studied in mice harboring floxed alleles of Eomes and expressing hematopoietic-restricted Cre recombinase under control of Vav regulatory elements (Eomes^flox/flox^Vav–Cre^+^ mice), which restricts the Eomes-inactivation to cells of the hematopoietic lineage (8). Deletion of Eomes resulted in a severe reduction of NK cells in spleen and blood whereas only a modest reduction in NK cell numbers was observed in liver, lymph node, and bone marrow. Deletion of Eomes and T-bet in Tbx21^−/−^Eomes^flox/flox^Vav–Cre^+^ mice resulted in complete absence of NK cells in all organs (8). Hence, T-bet and Eomes are essential for normal NK cell development, but in the absence of either T-bet or Eomes, an incomplete development may still occur suggesting that the two T-box transcription factors share several functions.

The analysis of the contribution of Eomes and T-bet to NK cell development also led to the identification of an ontologically distinct subset of innate lymphocyte (ILC) cells residing in the liver. Lymphocytes expressing NK cell markers in murine liver contain up to 40% of Eomes-negative cells that express high levels of T-bet (8, 9, 14). Hepatic Eomes^neg^T-bet^high^ NK cells display an immature phenotype characterized by the expression of Trail and lack of expression of DX5 (Trail^pos^DX5^neg^) (8). Initial experiments suggested that Eomes^neg^T-bet^high^Trail^pos^DX5^neg^ cells represented an intermediate developmental stage that could differentiate into mature Eomes^pos^Trail^neg^DX5^pos^ cells (8). However, experiments performed with Eomes-negative cells isolated from Eomes-GFP reporter mice demonstrated that Eomes^neg^T-bet^high^Trail^pos^DX5^neg^ cells are in fact an ontologically and functionally different subset of ILCs differentiating in the liver (14).

**Figure 1:**
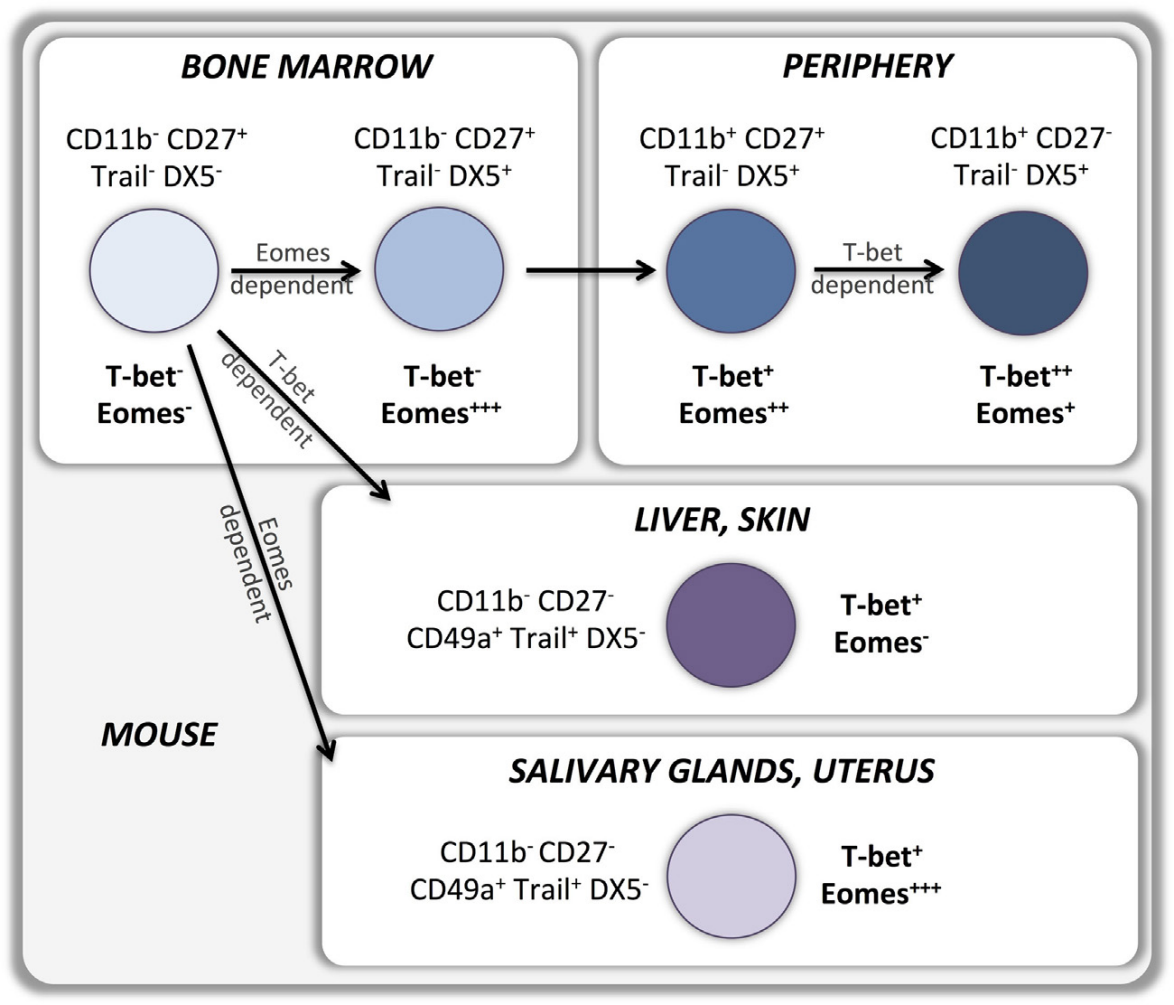
**T-bet and Eomes in murine NK cell development and peripheral maturation**.

**Figure 2:**
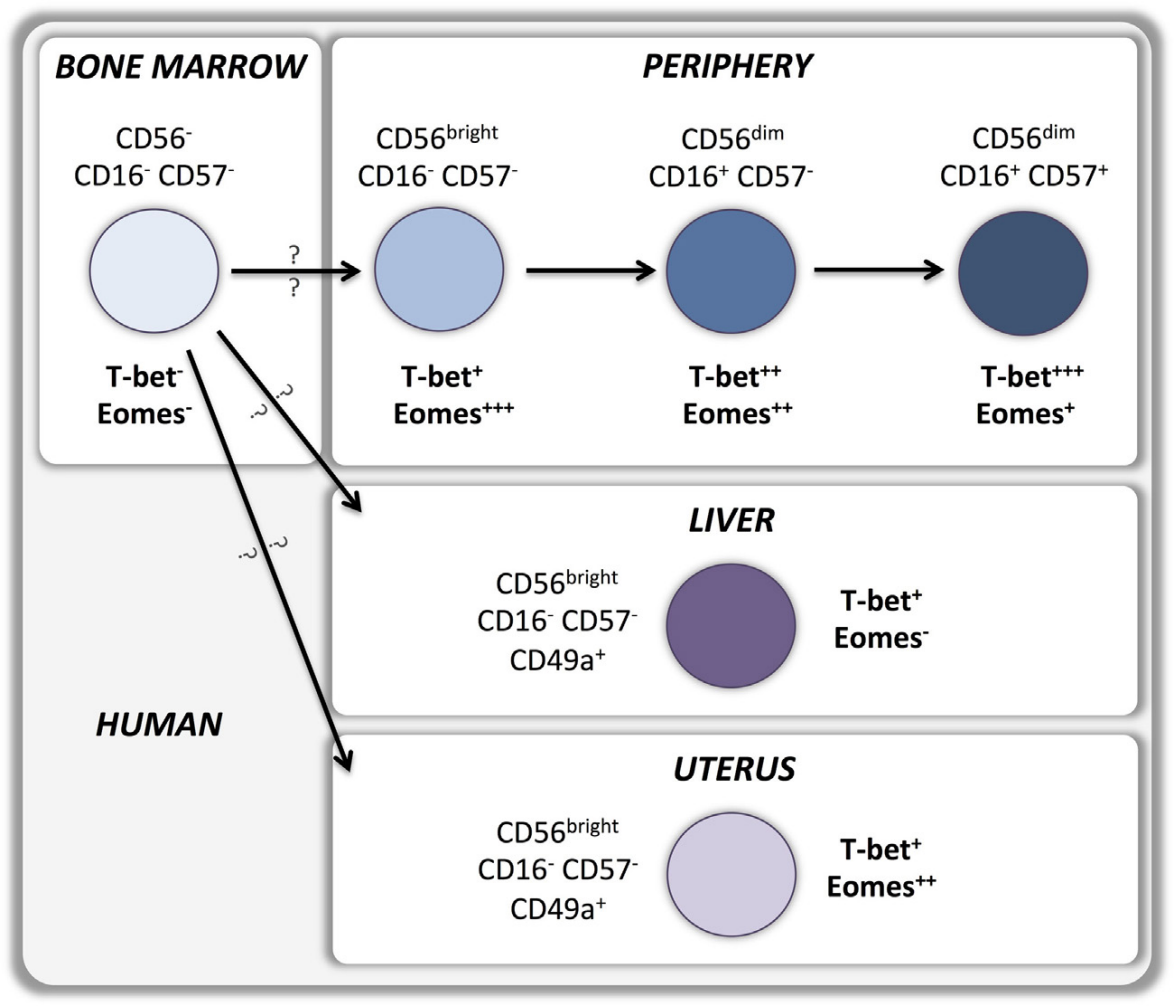
**T-bet and Eomes in human NK cell development and peripheral maturation**.

According to the current model, type 1 ILCs differentiate into two developmentally distinct lineages, type 1 helper innate lymphoid cells (hILC1s) and conventional NK cells (cNKs), which can be discriminated by the T-box transcription factors expressed (Figure 1). hILC1 differentiate in the liver when T-bet is upregulated and Eomes transcription is suppressed (14). Conversely, Eomes expression directs ILC1 development toward bone marrow-derived conventional NK cells that express relatively low levels of T-bet (15). Whether similar developmental pathways exist in human NK cells is still unknown (Figure 2). A recent study identified a T-bet^pos^Eomes^neg^ CD49a^pos^ NK cell subset in human liver, absent from hepatic venous or peripheral blood, with a CD56^bright^CD16^neg^CD57^neg^perforin^neg^ phenotype that may represent the human equivalent of murine T-bet^pos^ intrahepatic hILC1 (16).

In recent years, other ontologically distinct tissue residing NK cell subsets have been identified in salivary glands, skin, and uterus. Similarly to liver residing cells, NK cells isolated from these tissues display an immature CD49a^pos^DX5^neg^ phenotype associated with the expression of markers of tissue residency. However T-box transcription factors expression varies with the tissues the NK cells reside in, which may point at distinct developmental pathways. Skin residing NK cells do not express Eomes and strictly depend on T-bet for their development (17), suggesting a developmental relationship with liver residing NK cells. Conversely, salivary gland (18) and uterine (17, 19–21) NK cells express high levels of Eomes and are T-bet-independent for their development and appear therefore to be a more distinct NK cell lineage (Figure 1).

Little is known on the mechanisms that induce cells to upregulate or repress T-box transcription factors in different organs. By contrast, several cell intrinsic mechanisms regulating Eomes and T-bet expression have been elucidated to date. First, T-bet and Eomes regulate each other’s expression during NK cell development, and levels of Eomes expression correlate inversely with levels of T-bet in developing NK cells suggesting that active repressive mechanisms regulate the balance of T-bet/Eomes expression (14). This hypothesis has been confirmed by showing that T-bet^−/−^ NK cells express high levels of Eomes whereas transgenic NK cells overexpressing T-bet display low levels of Eomes (14). In addition, expression of T-bet and Eomes has been shown to be strictly related with expression of other transcription factors crucial for NK cell development. T-bet expression is induced by the transcription factors ETS1 (V-Ets Avian Erythroblastosis Virus E26 Oncogene Homolog 1) (22), TOX1 (thymocyte selection-associated HMG box protein TOX-1) (23), and TOX2 (24). Conversely, Eomes expression depends on the bZIP transcription factor Nfil3 (Nuclear factor, interleukin 3 regulated, also known as E4BP4), which binds to the regulatory regions of the Eomes gene to promote its transcription (25). Nfil3 deficiency interferes with the development of the Eomes^pos^TRAIL^neg^DX5^pos^ bone marrow-derived NK cells while hepatic Tbet^pos^Eomes^neg^TRAIL^pos^DX5^neg^ cells are unaffected (17, 26, 27). Interestingly, tissue-resident Eomes^pos^ NK cells localized in salivary glands and in the uterus can develop in the absence of Nfil3 (17, 18, 20, 27), suggesting the existence of alternative molecular mechanisms for the induction of Eomes transcription.

The only direct available information about the impact of T-box transcription factors deficiency on human NK cell development comes from a study describing a patient with a rare autosomal recessive microcephaly syndrome related to a translocation between chromosomes 3p and 10q leading to the silencing of the Eomes transcript (28). The fact that the infant displayed a normal distribution of T, B, and NK cells suggests that human NK cell development is possible in the absence of Eomes, although no information is available about the patient’s NK cell functionality.

## T-bet and Eomes in NK Cell Peripheral Maturation

Upregulation of T-bet and Eomes expression during development is maintained by most peripheral NK cells in mice (6) as well as in humans (10). Indeed, sustained expression of T-bet and Eomes in the periphery is necessary to maintain the NK cell maturation status while deletion of both T-box transcription factors results in reversion into an immature phenotype (8). T-bet expression is upregulated, and Eomes expression is downregulated during maturation of CD11b^pos^CD27^pos^ murine NK cells to the CD11b^pos^CD27^neg^ stage (14) (Figure 1). Importantly, T-bet appears to be essential for completion of this final maturation step as it controls the repression of CD27 and c-kit expression as well as the upregulation of S1P5 and KLRG1 (8, 13, 29–31). Therefore, bone marrow-derived Eomes^pos^TRAIL^neg^DX5^pos^ NK cells can develop in the absence of T-bet, but are unable to undergo the terminal stages of maturation. Part of this terminal maturation process seems to be mediated by T-bet induction of the PR domain zinc finger protein 1 (Blimp-1) (30) and the zinc finger E-box-binding homeobox 2 (ZEB2) (32) transcription factors. Conversely, Forkhead box protein O1 (FOXO1) inhibits NK terminal maturation through repression of T-bet (33). Similar patterns of T-bet and Eomes expression exist in human NK cells that also upregulate T-bet and downregulate Eomes during peripheral maturation (Figure 2). Cytokine-producing CD56^bright^ NK cells express higher levels of Eomes and lower levels of T-bet than cytotoxic CD56^dim^ NK cells (10–12, 34, 35). Moreover, terminally differentiated CD57^pos^CD56^dim^ NK cells express the highest levels of T-bet and the lowest levels of Eomes (11, 12). Accordingly, upregulation of Killer-cell immunoglobulin-like receptors (KIRs) during maturation is associated with a decrease of Eomes and an increase of T-bet levels (11), which appears to be independent of the fact whether KIRs are licensing or not (Pradier et al., unpublished observations submitted for the present Frontiers Immunology Research Topic).

## T-bet and Eomes in NK Cell Function

Chromatin Immunoprecipitation assays combined with the analysis of T-bet- and Eomes-deficient mice have partially uncovered the role of T-box transcription factors in NK cell biology. Similarly to what previously reported in CD4^+^ Th1 cells, ChIP experiments identified IFN-γ as a target gene of T-bet in NK cells (13). By contrast, no evidence of Eomes binding to the IFN-γ promoter has been reported. Conditions that induce IFN-γ production, such as stimulation with IL-12 plus IL-15 or with IL-12 plus IL-18, also induce upregulation of T-bet (8, 13) and Eomes (12). Murine NK cells are still able to produce IFN-γ *in vivo* in the absence of T-bet, Eomes, or both (8, 13), but the maintenance of IFN-γ production is impaired in the absence of T-bet (13). In addition, T-bet and Eomes expression correlates positively with IFN-γ production *in vitro* in mice (36) as well as in humans (12, 37, 38). Furthermore, NK cells are less cytotoxic in the absence of T-bet (13, 39), which is possibly caused by a decreased production of perforin and granzyme B (8, 13). Murine studies suggest that T-bet but not Eomes is directly involved in the production of cytotoxic molecules (8). Accordingly, we found a positive correlation between T-bet levels and perforin production in human NK cells and no relationship between Eomes levels and expression of cytotoxic molecules (12). Collectively, these findings support a model in which T-bet and Eomes cooperatively regulate IFN-γ production in NK cells while T-bet seems to be the crucial regulator of their cytotoxic activity.

## T-bet and Eomes in NK Cell Biology in Health and Disease

Given their impact in NK cell development, peripheral maturation and function, alterations in T-bet and Eomes expression could account for NK cell abnormalities in pathological conditions in which NK cells exert an essential role, such as infections and cancer. Reduction in T-bet and Eomes levels in NK cells occurs during aging and is associated with an impaired NK cell cytotoxicity (40). Interestingly, T-bet and Eomes downregulation in aged mice is not related to a cell intrinsic defect but is induced by the aged environment pointing to a cell extrinsic induction of a senescent phenotype.

The role of T-bet and Eomes expression in NK cells has been investigated in several disease models. NK cells activated during murine cytomegalovirus or vaccinia virus infection do not undergo terminal maturation in the absence of T-bet (13, 41, 42), which concords with the typical role of T-bet in NK cell differentiation (13). However, despite the fact that this led to a significant reduction of NK cell virus-specific cytotoxicity early after infection (13, 42), the viral load remained unchanged suggesting that the NK cell activity in T-bet^−/−^ mice is still sufficient to control viral replication. Similar sufficient *in vivo* NK cell responses have been reported after infection of T-bet^−/−^ mice with *Listeria monocytogenes* (43) or with *Toxoplasma Gondii* (44).

Murine models of cancer have illustrated the impact of T-box transcription factors in NK cell antitumor responses. Peng and coworkers used a transgenic prostate adenocarcinoma mouse model to demonstrate that although T-bet deficiency only had a very limited impact on primary tumor development, it significantly affected the ability to control tumor spread (45). This observation, subsequently confirmed in a murine model of metastatic cancer (46), led to the conclusion that *in vivo* NK activity in metastasized cancer strongly depends on T-bet expression. Although NK cells are still capable to infiltrate metastatic tumors in the absence of T-bet, their survival and capacity to terminally differentiate into fully competent, cytotoxic CD27^neg^KLRG1^pos^ NK cells appears to be diminished (31, 46). It has been show that *in vivo* IL-15 administration overcomes the defect of T-bet^−/−^ NK cells by inducing differentiation of Eomes^high^KLRG1^pos^ NK cells that are able to efficiently control metastatic pulmonary colorectal cancer, suggesting that IL-15 induced Eomes upregulation may compensate for the lack of T-bet inducing expansion of phenotypically and functionally mature NK cells.

Important insights into the relationship between T-bet and Eomes expression in NK cells and cancer come from the work of Gill and coworkers who identified the downregulation of T-bet and Eomes as the molecular signature of NK cell exhaustion in a murine NK adoptive transfer model of lymphoma (36). Importantly, downregulation of T-box transcription factors appeared to be not only the consequence of the NK cells’ exposure to tumor cells but also of their homeostatic proliferation induced by the treatment-induced lymphopenic environment. Lymphopenia occurs frequently after cancer chemotherapy as well as after conditioning regimens for hematopoietic stem cell transplantation (HSCT). Indeed, we found the same exhausted phenotype in human NK cells isolated from patients undergoing HSCT (12). Similar to what had been observed in mice, T-bet and Eomes downregulation after HSCT was associated with impaired NK function and lower levels of T-bet in NK cells were associated with reduced patient overall survival (12). Surprisingly, improved survival associated with higher levels of T-bet in NK cells was not the consequence of improved cancer control but the result of a reduced non-relapse mortality, which suggests that sustained T-bet and Eomes expression in NK cell could participate to prevent the development of transplant related complications after HSCT. This hypothesis is supported by a recent study showing that adoptively transferred IL-12/15/18-preactivated NK cells, which do not undergo exhaustion and maintain high levels of Eomes and T-bet expression, suppressed acute Graft-versus-Host-Disease in a murine model of HSCT (47). These results suggest that T-bet and Eomes expression may also modulate NK cell function in immunopathological settings, similarly to what recently shown in multiple sclerosis patients (35).

## Concluding Remarks

Recent findings clarifying the role of the two T-box transcription factors T-bet and Eomes in NK cells have considerably increased our knowledge of NK cell biology. Notably, they led to the characterization of previously unknown NK cells developmental pathways. Furthermore, they led to the identification of a molecular signature of NK cell exhaustion, which may represent a future target for immunomodulatory therapies.

## Author Contributions

FS wrote the manuscript and designed the figures. AP and ER critically discussed the work and edited the manuscript.

## Conflict of Interest Statement

The authors declare that the research was conducted in the absence of any commercial or financial relationships that could be construed as a potential conflict of interest.
